# Harnessing the duality of regulatory B cells: from identification of characteristic molecules to therapeutic targeting

**DOI:** 10.1093/immadv/ltag011

**Published:** 2026-06-26

**Authors:** Luman Wang, Ronghua Liu, Yiwei Chu

**Affiliations:** Department of Immunology, School of Basic Medical Sciences, Fudan University, Shanghai, China; Shanghai Fifth People’s Hospital, Fudan University, Shanghai, China; Shanghai Fifth People’s Hospital, Fudan University, Shanghai, China; Institutes of Biomedical Sciences, Fudan University, Shanghai, China; Department of Immunology, School of Basic Medical Sciences, Fudan University, Shanghai, China; Shanghai Fifth People’s Hospital, Fudan University, Shanghai, China

**Keywords:** Breg, phenotype, function, immunometabolism, applications

## Abstract

The field of immunology is increasingly intersecting with molecular biology, metabolic biology, and neurobiology (Zhu et al, A neuroimmune circuit mediates cancer cachexia-associated apathy. *Science* 2025;388:eadm8857; Jaschke et al, Gut-to-brain signaling restricts dietary protein intake during recovery from catabolic states. *Cell* 2025;188:7481–7494.e16; Zheremyan et al, Regulatory B cells: changing the landscape of immunoregulation and immunotherapy. *Cytokine Growth Factor Rev* 2025;86:121–36), marking a shift beyond traditional research approaches toward a more comprehensive understanding of the immune system. Central to this integrated perspective is the growing recognition that B cells are not only antibody producers, but also multifunctional cells dynamically modulated by their microenvironment. This comprehensive synthesis of recent research illuminates the dual nature of regulatory B cells (Bregs) as pivotal orchestrators of immune homeostasis. We examine how Bregs, governed by complex metabolic cues, neural signaling pathways, and local tissue microenvironments, can either suppress pathological inflammation or be co-opted to drive disease progression in autoimmunity, cancer, and allergic conditions. Understanding these microenvironmental determinants of Breg function is particularly critical for developing next-generation prognostic tools and targeted therapeutic strategies that effectively modulate B cell activity in various disease states.

## Introduction

From autoimmune diseases to cancer and allergic conditions, there is extensive interplay between tissue cells and immune cells; however, viewing the immune system in isolation has inherent limitations. Similar to how metabolic syndrome has been reconceptualized as an immunometabolic disorder, the functional capacity of immune cells demonstrates exquisite sensitivity to both systemic and local environmental signals [1]. Among these cellular mediators, regulatory B cells (Bregs) have emerged as particularly sophisticated integrators of diverse environmental signals [2]. While traditionally recognized for their production of anti-inflammatory cytokines including IL-10, TGF-β, and IL-35, Bregs maintain immune tolerance through multiple mechanisms while simultaneously exhibiting remarkable functional plasticity [3–5]. This special collection in Immunotherapy Advances brings together six original articles that redefine regulatory B cells (Bregs) as highly plastic, context-dependent immunomodulators shaped by metabolic, neural, and microenvironmental cues. Collectively, these studies show how systemic metabolic disturbances, local immunometabolic reprograming, and neuro-immune signaling can either reinforce Breg-mediated immune tolerance in inflammatory and allergic disease or be exploited to drive immunosuppression, therapeutic resistance, and disease progression in cancer and B-cell malignancies. By illuminating the mechanisms through which distinct tissue and disease contexts dictate B cell fate and function, this collection highlights emerging prognostic frameworks and therapeutic strategies that move beyond blanket B cell targeting toward precision interventions aimed at modulating specific suppressive or regulatory pathways.

## Metabolic and neural reprograming of B cell fate

Behçet's disease is a chronic systemic inflammatory condition with recurrent oral and genital ulcers, uveitis, and skin involvement. It serves as a vivid illustration of the fundamental interconnection between systemic metabolism and immune function, a cornerstone principle of modern immunometabolism. Lin and colleagues [6] demonstrate that elevated serum glucose and triglyceride levels function not merely as passive biomarkers but as active drivers of immune dysregulation. These circulating metabolites directly promote B cell activation, plasma cell differentiation, and enhanced antibody production, thereby positioning hyperglycemia and hypertriglyceridemia as central contributors to inflammatory pathology. These findings suggest that targeted metabolic interventions could serve as powerful adjuncts to conventional immunotherapies, potentially offering novel approaches to modulate aberrant immune responses in inflammatory conditions.

Beyond systemic metabolic influences, localized metabolic reprograming within tissue microenvironments can decisively direct B cell differentiation toward regulatory phenotypes. In allergic asthma models, Wang and colleagues describe a sophisticated feedback mechanism wherein M2 macrophage-derived TGF-β signaling shifts B cell metabolic programming toward oxidative phosphorylation (OXPHOS) [7]. This fundamental metabolic transition drives their subsequent differentiation into IL-10-producing Bregs, which effectively dampen inflammatory responses and contribute to tissue homeostasis restoration. This mechanism reveals a beneficial, metabolically orchestrated role for Bregs in resolving inflammation and highlights the potential for therapeutic strategies that target metabolic pathways to enhance regulatory immune function.

Furthermore, pathological microenvironments can actively hijack neural signaling pathways to induce immunosuppressive B cell responses. Gu and collaborators have uncovered a novel neuro-immune circuit in esophageal squamous cell carcinoma wherein nociceptive neurons release the neuropeptide CGRP (Calcitonin Gene-Related Peptide) [8]. Through signaling via the RAMP1 receptor expressed on B cells, CGRP drives their differentiation into a specialized regulatory subset characterized by TGF-β and IL-10 secretion. These neuro-modulated B cells directly promote CD8^+^ T cell exhaustion and confer significant resistance to anti-PD-1 immunotherapy, representing a previously unappreciated mechanism of treatment resistance in cancer therapy.

## Bregs as context-dependent mediators of disease

The functional consequences of Breg activity demonstrate remarkable context dependency across different disease states. In autoimmune conditions, Breg deficiency or functional impairment represents a recurring theme. As comprehensively reviewed by Lin and colleagues, Breg populations in multiple sclerosis and rheumatoid arthritis frequently exhibit both numerical deficiencies and functional impairments, characterized by inadequate IL-10 production and failure to suppress effector T cell responses, ultimately leading to breakdown of immunological tolerance. Paradoxically, in systemic lupus erythematosus, certain Breg populations may produce IL-10 that fuels autoantibody production, highlighting the complex and sometimes counterproductive roles these cells can play in different autoimmune contexts [9].

Conversely, within tumor microenvironments, Bregs are frequently co-opted to promote immunosuppression and facilitate disease progression. Research by Ge and colleagues [10] demonstrates that in clear cell renal cell carcinoma, a specific CD20 ^+^ CD23 ^+^ IL-10^+^ Breg subset fosters a profoundly immunosuppressive milieu characterized by impaired T cell activation and diminished innate immune responses. This pro-tumoral function, operational both within and outside tertiary lymphoid structures, significantly contributes to resistance against immune checkpoint blockade therapy. The development of a machine-learning-derived signature (CML Bregs) enables effective stratification of patients based on Breg activity levels and identifies the transcription factor IRF4 as a central regulatory node in this process, offering both prognostic utility and potential therapeutic targets.

B-cell malignancies similarly exploit microenvironment-driven immune resistance. Work by Blunt and collaborators [11] reveals that within lymph node niches, T cell-derived signals (CD40L, IL-4) upregulate expression of the inhibitory ligand HLA-E on malignant B cells. This surface molecule engages the NKG2A inhibitory receptor on natural killer (NK) cells, effectively blunting the efficacy of anti-CD20 antibody therapies—a compelling example of how local immune contexts can protect tumor cells from cytotoxic immune attacks.

## Therapeutic horizons: targeting B cells in context

The nuanced understanding of context-dependent Breg biology directly informs the development of novel therapeutic strategies that move beyond broad B cell depletion toward precision interventions targeting specific mechanistic pathways.

Reinforcing Breg function: In autoimmune and allergic conditions, therapeutic strategies aimed at boosting Breg numbers or enhancing their function show considerable promise. These approaches include pharmacological modulation using agents such as fingolimod, administration of natural immunomodulatory compounds derived from sources like Radix Rehmanniae, or sophisticated *ex vivo* expansion and reinfusion protocols for Breg populations [9].

Disrupting suppressive axes: In cancer immunotherapy, combining standard treatments with agents that specifically disrupt Breg-mediated suppression represents a crucial strategic direction. This includes blocking neuro-immune signaling with RAMP1/CGRP antagonists in esophageal squamous cell carcinoma, inhibiting the IRF4 pathway in clear cell renal cell carcinoma [8], or neutralizing the HLA-E/NKG2A checkpoint interaction in B-cell malignancies using anti-NKG2A antibodies [11].

Metabolic intervention: Targeting the metabolic drivers of pathogenic B cell activation, as suggested by findings in Behçet's disease, represents a complementary systemic approach that could potentially restore immune homeostasis by modifying the fundamental metabolic environment that shapes B cell behavior [6].

## Conclusion and future perspectives

The collective evidence firmly positions B cells, particularly Bregs, as central processors of immunological context, integrating diverse signals from metabolic, neural, and immunological environments to determine functional outcomes. The future of immunotherapy fundamentally depends on embracing this complexity and moving from a static view of B cells to a dynamic perspective that accounts for how their function is continuously shaped within specific disease microenvironments. Realizing this vision will require several key advancements:

Advanced profiling technologies: Implementation of multi-omics approaches and spatial transcriptomics to define context-specific Breg signatures and identify novel subpopulations with distinct functional capabilities across different disease states.

Mechanistic elucidation: Deeper investigation into the molecular mechanisms through which metabolites, neuropeptides, and cell–cell contacts reprogram B cell fate and function, with particular emphasis on signaling pathway integration and epigenetic modifications.

Innovative combination therapies: Strategic design of clinical trials that rationally pair conventional immunotherapies with agents that target the specific pathways co-opting Breg function in particular disease contexts, with careful attention to timing, sequencing, and patient stratification.

By developing the capability to precisely manipulate the contextual switches that control B cell function, we can potentially harness their dual nature to suppress autoimmunity, counteract allergic inflammation, and dismantle tumor defenses, ultimately restoring immune equilibrium across a broad spectrum of human diseases. This integrative approach represents not merely an incremental improvement in existing paradigms, but a fundamental reimagining of B cell biology that acknowledges their role as sophisticated environmental sensors and responsive immunomodulators within the complex ecosystems of health and disease.

## Data Availability

None declared.

## References

[1] Rosser EC, Mauri C. The emerging field of regulatory B cell immunometabolism. Cell Metab 2021;33:1088–97. 10.1016/j.cmet.2021.05.008 34077716

[2] Jansen K, Cevhertas L, Ma S et al Regulatory B cells, A to Z. Allergy 2021;76:2699–715. 10.1111/all.14763 33544905

[3] Zheng H, Cao P, Su Z et al Insights into the roles of IL-10-producing regulatory B cells in cardiovascular disorders: recent advances and future perspectives. J Leukoc Biol 2023;114:315–24. 10.1093/jleuko/qiad066 37284816

[4] Jing R, Liao X, Mo J et al Spatiotemporal regulation of ventilator lung injury resolution by TGF-β1 + regulatory B cells via macrophage vesicle-nanotherapeutics. Front Immunol 2025;16:1635178. 10.3389/fimmu.2025.1635178 40709179 PMC12286806

[5] Saheb Sharif-Askari F, Zakri AM, Alenazy MF et al IL-35 promotes IL-35(+)IL-10(+) Bregs and conventional LAG3(+) tregs in the lung tissue of OVA-induced asthmatic mice. Inflamm Res 2024;73:1699–709. 10.1007/s00011-024-01925-1 39127869

[6] Li M, Li P, Wang X et al Abnormal glucose and lipid metabolism promotes disrupted differentiation of T and B cell subsets in Behçet's disease. Immunother Adv 2025;5:ltaf010. 10.1093/immadv/ltaf010 40297266 PMC12036013

[7] Yu B, Wang X, Zheng Y et al M2 macrophages promote IL-10(+)B-cell production and alleviate asthma in mice. Immunother Adv 2025;5:ltaf007. 10.1093/immadv/ltaf007 40342727 PMC12059559

[8] Zhang H, Zhang Y, Zhou P et al Intra-tumoural RAMP1+ B cells promote resistance to neoadjuvant anti-PD-1-based therapy in oesophageal squamous cell carcinoma. Immunother Adv 2025;5:ltaf012. 10.1093/immadv/ltaf012 40385640 PMC12084762

[9] Yan H, He J, Lin X. Heterogeneity of regulatory B cells in autoimmune diseases: implications for immune equilibrium and therapeutic strategies. Immunother Adv 2025;5:ltaf020. 10.1093/immadv/ltaf020 40458534 PMC12128196

[10] Ge Q, Zhou S, Lu J et al Regulatory B cells promote the immunosuppressive microenvironment and progression of clear cell renal cell carcinoma. Immunother Adv 2025;5:ltaf013. 10.1093/immadv/ltaf013 40575014 PMC12202094

[11] Graham LV, Horehajova L, Haselager MV et al CD40L and IL-4 suppress NK cell-mediated antibody-dependent cellular cytotoxicity through the HLA-E:NKG2A axis. Immunother Adv 2025;5:ltaf029. 10.1093/immadv/ltaf029 40977848 PMC12448733

